# Breast Cancer–Specific Survival and Prognostic Factors in a Statewide Oncology Network in Brazil: A Registry‐Linked Retrospective Cohort Study

**DOI:** 10.1002/cnr2.70467

**Published:** 2026-01-29

**Authors:** Raphael Manhães Pessanha, Wesley Rocha Grippa, Luiz Cláudio Barreto Silva Neto, Naira Santos D'Agostini, Luís Carlos Lopes‐Júnior

**Affiliations:** ^1^ Graduate Program in Public Health (PPSCG), Health Sciences Center Universidade Federal do Espírito Santo (UFES) Vitória Espírito Santo Brazil; ^2^ Pan American Health Organization Washington DC USA

**Keywords:** breast neoplasms, Kaplan–Meier estimate, proportional hazards models, survival analysis

## Abstract

**Background:**

Breast cancer survival varies widely across middle‐income settings and may be influenced by clinical stage at diagnosis and access pathways within oncology care networks. In Brazil, evidence from statewide cohorts using linked registry and mortality data remains limited.

**Aim:**

To investigate the association between clinical factors and breast cancer–specific survival among women treated in all hospitals comprising the Oncology Care Network of a state in Southeastern Brazil.

**Methods and Results:**

A retrospective cohort study was conducted using data from the Hospital Cancer Registry linked to the state Mortality Information System. Women aged ≥ 18 years diagnosed with primary breast cancer (ICD‐10: C50.x) between 2000 and 2016 were included and followed until December 31, 2021. Five‐year breast cancer–specific survival was estimated using the Kaplan–Meier method, and factors associated with mortality were assessed using cause‐specific Cox proportional hazards models with complete‐case analysis. A total of 12,096 women were included, of whom 7,191 had complete data for multivariable analysis. The mean age at diagnosis was 54.7 years. Five‐year breast cancer–specific survival ranged from 97% (95% CI: 96−97%) in stage I to 32% (95% CI: 31−37%) in stage IV. Women referred from the private healthcare system had a significantly lower risk of breast cancer mortality than those referred from the public system (HR: 0.83; 95% CI: 0.75−0.93; *p* = 0.001). Advanced clinical stage remained the strongest predictor of mortality, and the presence of distant metastasis at diagnosis increased the risk of breast cancer death by 49% (HR: 1.49; 95% CI: 1.14−1.94; *p* = 0.003).

**Conclusion:**

Breast cancer–specific survival in Espírito Santo is strongly determined by stage at diagnosis and by differential access pathways within the oncology care network. Strengthening early diagnostic strategies, improving referral coordination, and ensuring equitable access to timely treatment are essential to reduce survival disparities in this setting.

## 1 Introduction

Breast cancer remains the most common cancer in women worldwide [1, 2, 3] According to the most recent GLOBOCAN 2022 estimates, approximately 2.3 million new cases of breast cancer and 670 000 deaths occurred globally in 2022, reaffirming breast cancer as the leading cause of cancer‐related morbidity and mortality among women across most world regions [2, 3]. Incidence rates continue to be highest in North America, Western Europe, and Oceania [2, 3]. In the United States, updated projections estimate nearly 310 000 new cases of invasive breast cancer and more than 43 000 deaths, underscoring the persistent public health burden of the disease in high‐income settings [1].

In Brazil, 73 610 new cases of breast cancer were estimated in 2023, corresponding to an incidence of 66.54 cases per 100 000 women [4]. The Southeast region shows the highest incidence (84.46/100000), followed by the South, Central‐West, Northeast, and North regions of Brazil [4]. Breast cancer is also the leading cause of cancer‐related mortality among Brazilian women, with the Southeast presenting the highest mortality burden [5]. These regional differences reflect persistent disparities in timely diagnosis, early detection, and access to appropriate treatment, resulting in heterogeneous survival outcomes across the country [6, 7].

Previous studies have evaluated breast cancer survival in Brazil using hospital‐based, municipal or multicenter cohorts [8, 9] analyzed 2715 cases from a single municipality and reported improved survival following expanded mammography coverage; however, the study reflects the characteristics of a localized population with a well‐established screening program and does not capture the broader heterogeneity of state‐wide oncology networks. Likewise, Ferreira et al. [8] compared survival between public and private care in a referral hospital and found higher survival in the private sector (80.6% vs. 68.5% at 5 years), but their single‐center design and absence of linkage with mortality registries limit representativeness and may underestimate deaths occurring outside the institution. Collectively, these studies—while informative—do not reflect the structural complexity, referral pathways, or service distribution of entire state‐level Oncology Care Networks, underscoring the need for broader, population‐level analyses using integrated data sources.

Espírito Santo presents a particularly relevant context for breast cancer survival research. Its Oncology Care Network is characterized by pronounced public–private segmentation, heterogeneous service distribution, and variable referral pathways across municipalities [10]. Nearly half of the population relies exclusively on the Unified Health System (SUS), while the remainder accesses private healthcare, resulting in complex, mixed care‐seeking behavior and differential access to diagnostic and therapeutic services. Furthermore, specialized oncology services are disproportionately concentrated in the metropolitan region of the capital, which can contribute to diagnostic delays, prolonged referral times, and potential stage migration among women residing in inland or rural municipalities. These organizational, geographic, and sociocultural characteristics distinguish Espírito Santo from other Brazilian states and justify the need for a comprehensive, statewide survival analysis [10].

Hospital Cancer Registries (HCR) offer real‐world data essential for monitoring cancer outcomes, and their linkage with mortality databases enables more accurate estimation of cancer‐specific survival and evaluation of prognostic factors. However, so far, in Espírito Santo, Brazil, most evaluations involving HCR [11, 12, 13, 14, 15], have focused primarily on data completeness and information quality, or on survival analyses of tumors other than breast cancer, rather than on linkage‐based approaches. Despite maintaining established HCR, Espírito Santo lacks integrated analyses combining HCR data with the Mortality Information System (Sistema de Informação de Mortalidade—SIM). The absence of linkage between these systems limits the ability to generate accurate, population‐based estimates of breast cancer–specific survival and restricts the evaluation of prognostic factors across the entire care network. Although linkage‐based methodologies have shown strong potential to improve cancer surveillance and enhance the accuracy of survival estimates in Brazil, such integrated analyses remain scarce in the state of Espírito Santo.

This study addresses this gap by linking the Hospital Cancer Registry (HCR) with the state Mortality Information System (SIM) to estimate breast cancer–specific survival within the Oncology Care Network of Espírito Santo from 2000 to 2016. We examined prognostic factors related to referral source, clinical stage, metastatic status, histologic type, and initial treatment modalities. By leveraging a large, statewide cohort encompassing both public and private sectors, this study provides new real‐world evidence on survival disparities in a middle‐income setting and offers insights to guide cancer control strategies, health service organization, and public policy planning in Brazil.

Hence, the objective of this study was to investigate the association between clinical factors and the survival of women diagnosed with breast cancer in all hospitals that make up the Oncology Care Network of a southeastern Brazilian state between 2000 and 2016.

## 2 Methods

### 2.1 Study Design

This is a retrospective, hospital‐based cohort study conducted within the Oncology Care Network of Espírito Santo, Brazil. The study used secondary data from the Hospital Cancer Registry (HCR) of Espírito Santo, Brazil and the state Mortality Information System (Sistema de Informação de Mortalidade—SIM). The study was reported as follows: the recommendations of the STROBE Statement for cohort studies [16].

### 2.2 Setting

The Oncology Care Network of Espírito Santo covers three health regions: North/Central, Metropolitan and South [11]. Cancer care in the state is organized through one High‐Complexity Oncology Center (CACON) and seven High‐Complexity Oncology Units (UNACON), all accredited by the Brazilian Ministry of Health. The CACON is represented by Afecc‐Hospital Santa Rita de Cássia, located in the state capital, Vitória, and functions as the main referral center for oncology services. The seven UNACONs are geographically distributed across the state: Hospital Evangélico de Cachoeiro de Itapemirim (Cachoeiro de Itapemirim, ES, Brazil); Hospital Evangélico de Vila Velha (Vila Velha, ES, Brasil); Hospital Universitário Antônio Cassiano de Moraes, Santa Casa de Misericórdia de Vitória, and Hospital Estadual Infantil Nossa Senhora da Glória (all located in Vitória, ES, Brazil); Hospital São José (Colatina, ES, Brazil); and Hospital Rio Doce Hospital (Linhares, ES, Brazil). These oncology units routinely provide diagnosis, treatment, and follow‐up for breast cancer patients, and continuously submit standardized records to the national Integrating System (SIS‐RHC), ensuring systematic reporting and data quality for hospital‐based cancer surveillance [14, 17].

### 2.3 Participants

Women aged 18 years or older with a primary diagnosis of malignant breast cancer (ICD‐10: C50.0–C50.9), confirmed by pathology, who received care in any hospital within the Oncology Care Network of Espírito Santo and were registered in the national Hospital Cancer Registry system (SIS‐RHC) between January 1, 2000, and December 31, 2016, were eligible for inclusion. Only residents of Espírito Santo were considered for this cohort.

#### 2.3.1 Inclusion Criteria

1. Female sex.
2. Age ≥ 18 years at diagnosis
3. Pathologically confirmed malignant breast cancer (ICD‐10: C50.0–C50.9).
4. Registered in the SIS‐RHC during the study period (2000–2016).
5. Successful deterministic linkage to the Mortality Information System (SIM) or classification as alive at last follow‐up.

#### 2.3.2 Exclusion Criteria

1. Male patients.
2. Duplicate records identified during data cleaning.
3. Missing or invalid information for key variables (clinical stage, metastatic status, referral source, vital status, or date fields).

#### 2.3.3 Final Cohort

After applying all eligibility criteria, 12 096 women composed the final analytic sample used for descriptive statistics and survival analyses. The complete‐case dataset used in the multivariable Cox regression included 7191 women with breast cancer.

#### 2.3.4 Follow‐Up

Follow‐up began at the date of breast cancer diagnosis and ended at the earliest of:

- date of breast cancer–specific death,
- date of death from other causes, or
- December 31, 2021 (administrative censoring).

### 2.4 Variables and Outcomes

Information on sociodemographic, clinical, and treatment‐related variables was obtained from the HCR. The clinical variables used in this study derive from the standardized Tumor Registration Form of the SIS‐RHC, a validated instrument developed by the Brazilian National Cancer Institute (INCA) and recognized by the International Agency for Research on Cancer (IARC/WHO). The HCR is a structured system responsible for the systematic and continuous collection, storage, processing, and dissemination of information on patients treated for malignant tumors in accredited hospital units [18]. Its standardized registration form contains 44 items, including sociodemographic, clinical, tumor‐related, treatment, and outcome variables—35 mandatory and nine optional—which ensure uniformity and comparability of cancer data [18].

From this form, the following variables were selected for the present analysis: age at diagnosis, referral source, previous diagnosis or treatment, diagnostic basis, histological type of the primary tumor, presence of multiple primary tumors, TNM staging, distant metastasis, first treatment received in the hospital, treatment status at the end of the first hospital course, family history of cancer, tumor laterality, reasons for not receiving antineoplastic treatment, and clinical outcome [18].

The main exposure variable was the referral source, categorized as the Public healthcare system (SUS) or the Private healthcare system. Clinical variables of interest included the tumor stage at diagnosis, originally recorded following TNM criteria and subsequently grouped into stages I, II, III, and IV; metastatic status at presentation (yes/no); histologic type, classified as ductal, lobular, or other subtypes; and initial treatment modalities, including surgery, chemotherapy, radiotherapy, and hormone therapy, each coded as present or absent. Additional covariates included age at diagnosis (continuous), education level, municipality of residence (metropolitan vs. non‐metropolitan), and year of diagnosis, categorized into predefined temporal intervals.

The primary outcome was breast cancer–specific mortality, defined as death for which a malignant neoplasm of the breast (ICD‐10: C50.x) was recorded as the underlying cause in the Mortality Information System (SIM). Time‐to‐event was calculated from the date of pathological diagnosis in the HCR to the date of breast cancer death. Women who remained alive at the end of follow‐up or who died from causes unrelated to breast cancer were censored at the date of last known vital status.

## 3 Data Sources and Measurement

Data for this study were obtained from the HCR and the Mortality Information System of Espírito Santo (SIM/ES). To ensure complete ascertainment of vital status and cause of death, we performed a deterministic record linkage between the two databases. Before linkage, both datasets underwent standard data‐cleaning procedures, including removal of duplicate entries and harmonization of key identifiers (name fields, date formats, sex, and municipality of residence). Deterministic linkage, a method that integrates multiple data sources based on exact agreement of identifiers, was applied according to predefined matching criteria [19].

Record linkage was performed using exact agreement across core identifiers—patient's name, mother's name, date of birth, sex, and municipality of residence. When necessary, potential matches were manually reviewed using additional variables such as full residential address and dates of diagnosis and death. Only record pairs with complete concordance across required identifiers were retained. This approach ensured accurate identification of breast cancer–specific deaths not captured in the hospital registry and updated the vital status of all eligible women.

Data extraction occurred between June and October 2023, and the linkage procedures were completed in December 2023.

### 3.1 Data Analysis

Descriptive analyses were used to summarize sociodemographic and clinical characteristics. Categorical variables were presented as absolute frequencies and percentages, while continuous variables were summarized using means, medians, and standard deviations. Breast cancer–specific survival was estimated using the Kaplan–Meier method [20], and differences between survival curves were assessed using the log‐rank test [21].

To identify factors associated with breast cancer–specific mortality, we fitted a cause‐specific Cox proportional hazards model, in which deaths due to breast cancer (ICD‐10: C50.x) were treated as events and deaths from other causes were censored [22]. Variables showing statistical significance in univariate analyses and satisfying the proportional hazards assumption were considered for multivariable modelling. The final model included referral source, previous diagnosis and treatment, clinical stage, metastatic status, and receipt of initial treatment modalities.

All women registered in the HCR/SIM with a breast cancer diagnosis between 2000 and 2016 were included in the descriptive and survival analyses, and all individuals had a minimum potential follow‐up of 5 years (censoring date: December 31, 2021). A complete‐case analysis was conducted prior to modelling. Cases with missing or invalid information for clinical stage, metastatic status, referral source, vital status, or dates were excluded, yielding a final multivariable sample of *n* = 7191 women.

Cases with missing stage information were excluded from multivariable analyses because stage was not missing at random. In the HCR, absence of staging reflects heterogeneous diagnostic pathways and reporting practices rather than a biologically meaningful category. Creating an ‘unknown stage’ group would therefore introduce misclassification bias and impair interpretability of hazard ratios. Consistent with STROBE recommendations, a complete‐case approach was used for multivariable modelling.

Multiple imputation was not applied because missingness was concentrated in variables with structural or non‐random patterns (e.g., family history of cancer and detailed treatment combinations), which violated key assumptions required for valid imputation. In addition, several variables—including histologic subtype details, presence of multiple primary tumors, and specific multimodal treatment combinations (e.g., Surgery (SX) + chemotherapy (CT) + radiotherapy (RT), surgery (SX) + chemotherapy (CT) + radiotherapy (RT) + hormone therapy (HT) and other combinations)—did not satisfy the proportional hazards assumption and were therefore excluded from the multivariable analysis.

In addition, treatment variables (surgery, chemotherapy, radiotherapy, and hormone therapy) were modeled as categorical indicators of whether each modality was received during first‐course treatment, without incorporating treatment initiation dates. Because treatment timing was not available in analyzable form for all patients, time‐dependent modelling was not possible, and we acknowledge that this may introduce potential immortal time bias when interpreting associations between treatment and breast cancer–specific mortality. Therefore, treatment variables were interpreted descriptively and not as causal estimates of treatment effectiveness.

The variable laterality was also excluded due to extreme imbalance between categories (unilateral: 7113; bilateral: 78), which prevented stable model estimation.

Statistical analyses were performed using R (version 4.1.0) and RStudio (version 2022.07.2), assuming a two‐sided significance level of 5%.

### 3.2 Ethical Aspects

The research project was approved by the Research Ethics Committee of the Federal University of Espírito Santo (opinion number: 3831617). Additionally, the Espírito Santo State Health Department (SESA) granted approval and authorization for the collection of secondary and restricted institutional data pertinent to the study. Because this study used only de‐identified secondary data from the Hospital Cancer Registry (HCR) and the Mortality Information System (SIM), the requirement for individual informed consent was waived by the Research Ethics Committee of the Federal University of Espírito Santo (approval no. 3831617), in accordance with national regulations and the principles of the Declaration of Helsinki.

## 4 Results

A total of 12 096 cases of women diagnosed with breast cancer in the Oncology Care Network of ES during the study period were analyzed. Table 1 presents the baseline characteristics of the selected sample. The mean age of the women was 54.67 years (SD = 13.10). Most of the sample of women diagnosed with breast cancer were referred through the Unified Health System (SUS) (57.30%), had no previous diagnosis or treatment (43.11%), and the histology of the primary tumor was the most important basis for the diagnosis (96.49%).

**TABLE 1:** Baseline characteristics of participants.

Variable	*N*	%
Age at diagnosis (in years)		
Mean (standard deviation)	54.67 (13.10)	—
Median (interquartile range)	53 (45–64)	—
Forwarding source		
Public healthcare system (SUS)	6931	57.30
Private healthcare system	3118	25.78
Missing data	2047	16.92
Previous diagnosis and treatment		
No Diagnosis/no treatment	5215	43.11
With Diagnosis/no treatment	2968	24.54
With diagnosis/withtreatment	3788	31.32
Other	64	0.53
Missing data	61	0.50
Most important basis for tumor diagnosis		
Histology of the primary tumor	11 671	96.49
Imaging examination	256	2.12
Other	114	0.94
Missing data	55	0.45
Histological type of primary tumor		
Invasive carcinoma of no special type	9103	75.26
Ductal carcinoma in situ	848	7.01
Invasive lobular carcinoma	709	5.86
Other	1436	11.87
Occurrence of more than one primary tumor		
No	11 328	93.65
Yes	538	4.45
Uncertain	33	0.27
Missing data	197	1.63
Clinical tumor staging by group (TNM)		
Stage 0 (in situ)	501	4.14
Stage I	2032	16.80
Stage II	3338	27.60
Stage III	2184	18.06
Stage IV	1001	8.28
Missing data	3040	25.13
Distant metastasis		
No	11 052	91.37
Yes	1044	8.63
First treatment received at the hospital		
No treatment	511	4.22
Surgery (SX)	1311	10.84
Radiotherapy (RT)	1351	11.17
Chemotherapy (CT)	597	4.94
Hormone therapy (HT)	646	5.34
SX + RT	546	4.51
CT + SX	780	6.45
SX + CT + RT	1078	8.91
SX + CT + RT + HT	1031	8.52
Other combinations	4193	34.66
Missing data	52	0.43
Disease status at the end of the first hospital treatment		
No evidence of disease (complete remission)	2186	18.07
Partial remission	213	1.76
Stable disease	1631	13.48
Disease progression	407	3.36
Oncologic supportive care	215	1.78
Death	1028	8.50
Missing data	6416	53.04
Family history of cancer		
No	3372	27.88
Yes	2571	21.25
Missing data	6153	50.87
Tumor laterality		
Unilateral	11 300	93.42
Bilateral	143	1.18
Missing data	653	5.40
Outcomes		
Survived at end of follow‐up	8184	67.66
Died of breast cancer	2937	24.28
Died from other causes	975	8.06

Invasive carcinoma was the most common histological type (75.26%), and there was no occurrence of more than one primary tumor (93.65%). Stage II was the most prevalent (27.60%), followed by III (18.06%), I (16.60%), and IV (8.28%), and the majority did not present distant metastasis (91.37%). Among patients who received only one type of treatment, radiotherapy was the most frequent (11.17%), followed by surgery (10.84%), hormone therapy (5.34%), and chemotherapy (4.94%). Among patients who received multimodal treatments, the most frequent were: The most common combinations of treatments were SX + CT + RT (8.91%), SX + CT + RT + HT (8.52%), CT + SX (6.45%), and SX + RT (4.51%).

In relation to the initial treatment received at the hospital, the majority (34.66%) of patients received other combinations of previous treatments. At the end of initial hospital treatment, 18.07% had complete remission, 13.48% had stable disease, and 8.50% succumbed to their illness. Among the registered cases, 21.25% had a family history of cancer, and the majority had a unilateral tumor (93.42%).

Regarding the outcome, the majority of patients survived until the end of the follow‐up period (67.66%), although 24.28% died as a result of breast cancer and 8.06% died from other causes. It was observed that the variables “TNM,” “family history of cancer,” “disease status at the end of the first hospital treatment,” and “main reason for not carrying out antineoplastic treatment in the hospital” exhibited high percentages of incompleteness (missing) (25.13%, 50.87%, 53.04%, and 83.01%, respectively).

Table 2 presents survival estimates over a five‐year follow‐up period, employing the Kaplan–Meier method. The overall survival of the sample was 82.4% (95% CI: 0.81–0.83). The group with Private healthcare system referral origin exhibited a higher overall survival (OS = 0.88; 95% CI: 0.87–0.89; *p* < 0.001) compared to those who were referred by the SUS (OS = 0.79; 95% CI: 0.78–0.80; *p* < 0.001). The group with a previous diagnosis and treatment exhibited a higher overall survival rate (OS = 0.89; 95% CI: 0.88–0.90; *p* < 0.001) compared to those who did not have a previous diagnosis and treatment (OS = 0.80; 95% CI: 0.78–0.81; *p* < 0.001).

**TABLE 2:** Overall survival (OS) and 95% confidence interval (95% CI) estimated by the Kaplan–Meier method for breast cancer mortality at 5 years.

Variable	*N*	5‐year OS^a^		Log‐rank test
		OS	95% CI	*p* value
Overall population	12.09	0.82	0.81–0.831	—
Forwarding source	10.04	—	—	< 0.001
Public healthcare system (SUS)	6.93	0.79	0.78–0.80	—
Private healthcare system	3.11	0.88	0.87–0.89	—
Previous diagnosis and treatment	11.97	—	—	< 0.001
No Diagnosis/no treatment	5.21	0.80	0.78–0.81	—
With Diagnosis/no treatment	2.96	0.77	0.76–0.79	—
With Diagnosis/with treatment	3.78	0.89	0.88–0.90	—
Histological type of primary tumor^b^	12.09	—	—	< 0.001
Invasive carcinoma of no special type	9.10	0.80	0.79–0.81	—
Ductal carcinoma in situ	848	0.97	0.96–0.98	—
Invasive lobular carcinoma	709	0.81	0.78–0.84	—
Other	1.43	0.85	0.83–0.87	—
Occurrence of more than one primary tumor^b^	11.86	—	—	0.030
Yes	11.32	0.82	0.81–0.82	—
No	538	0.87	0.84–0.90	—
Clinical tumor staging by group (TNM)	9.05	—	—	< 0.001
Stage 0 (in situ)	501	0.99	0.99–1.00	—
Stage I	2.03	0.97	0.96–0.97	—
Stage II	3.33	0.89	0.88–0.90	—
Stage III	2.18	0.69	0.67–0.71	—
Stage IV	1.00	0.32	0.29–0.35	—
Distant metastasis	12.09	—	—	< 0.001
No	11.05	0.86	0.86–0.87	—
Yes	1.04	0.34	0.31–0.37	—
Family history of cancer^b^	5.94	—	—	< 0.001
No	3.37	0.79	0.78–0.80	—
Yes	2.57	0.83	0.82–0.84	—
Tumor laterality	11.44	—	—	0.002
Unilateral	11.30	0.82	0.81–0.83	—
Bilateral	143	0.72	0.65–0.80	—
Surgery (SX)	12.09	—	—	< 0.001
No	10.78	0.82	0.81–0.82	—
Yes	1.31	0.86	0.84–0.87	—
Radiotherapy (RT)	12.09	—	—	< 0.001
No	10.74	0.81	0.80–0.82	—
Yes	1.35	0.89	0.87–0.90	—
Chemotherapy (CT)	12.09	—	—	< 0.001
No	11.49	0.83	0.82–0.84	—
Yes	597	0.61	0.57–0.65	—
Hormone therapy (HT)	12.09	—	—	< 0.001
No	11.45	0.81	0.81–0.82	—
Yes	646	0.93	0.91–0.95	—
SX + RT	12.09	—	—	< 0.001
No	11.55	0.82	0.81–0.82	—
Yes	546	0.91	0.89–0.94	—
CT + SX	12.09	—	—	< 0.001
No	11.31	0.82	0.82–0.83	—
Yes	780	0.74	0.71–0.78	—
SX + CT + RT^b^	12.09	—	—	0.008
Yes	11.01	0.82	0.81–0.83	—
No	1.07	0.79	0.77–0.82	—
SX + CT + RT + HT^b^	12.09	—	—	0.9
No	11.06	0.82	0.81–0.82	—
Yes	1.03	0.86	0.84–0.89	—
Other combinations^b^	12.09	—	—	0.001
No	7.90	0.83	0.82–0.83	—
Yes	4.19	0.81	0.80–0.82	—

^a^ Overall survival (OS) and 95% confidence interval (95% CI) estimated by the Kaplan–Meier method for breast cancer mortality.

^b^ Not included in the Cox multivariate analysis due to non‐proportional risks over time, as shown in Figure 1.

Concerning the histological type of the primary tumor, ductal carcinoma in situ exhibited the highest overall survival rate over a five‐year period (OS = 0.97; 95% CI: 0.96–0.98; *p* < 0.001), whereas invasive breast carcinoma demonstrated the lowest 5‐year survival rate (OS = 0.80; 95% CI: 0.79–0.81; *p* < 0.001). The overall survival of women with more than one primary tumor was found to be lower than that of women without tumors (OS = 0.82; 95% CI: 0.81–0.82) (*p* = 0.03).

The results demonstrated that patients with stage I (OS = 0.97; 95% CI: 0.96–0.97) and stage II (OS = 0.89; 95% CI: 0.88–0.90) had greater survival compared to those with advanced stage III (OS = 0.69; 95% CI: 0.67–0.71) and stage IV (OS = 0.32; 95% CI: 0.29–0.35). These findings indicated a statistically significant difference (*p* < 0.001).

The five‐year survival rate for women without distant metastasis was found to be significantly higher than that of women with metastasis (OS = 0.86; 95% CI: 0.86–0.87; *p* < 0.001). Women with a single tumor exhibited a longer survival period (OS = 0.82; 95% CI: 0.81–0.83; *p* = 0.002) in comparison to those with bilateral tumors (OS = 0.72; 95% CI: 0.65–0.80; *p* = 0.002).

With regard to those who received only one of the isolated treatments, survival exhibited variability between those who did and did not receive it. Those who underwent surgical treatment exhibited greater survival (OS = 0.86; 95% CI: 0.84–0.87; *p* < 0.001) compared to those who did not (OS = 0.82; 95% CI: 0.81–0.82; *p* < 0.001). Those who underwent radiotherapy exhibited greater survival (OS = 0.89; 95% CI: 0.87–0.90; *p* < 0.001) compared to those who did not receive this treatment (OS = 0.81; 95% CI: 0.80–0.82; *p* < 0.001). About multimodal treatments, the survival rates observed for the various therapeutic combinations presented in Table 2 also exhibited notable variability.

**FIGURE 1:**
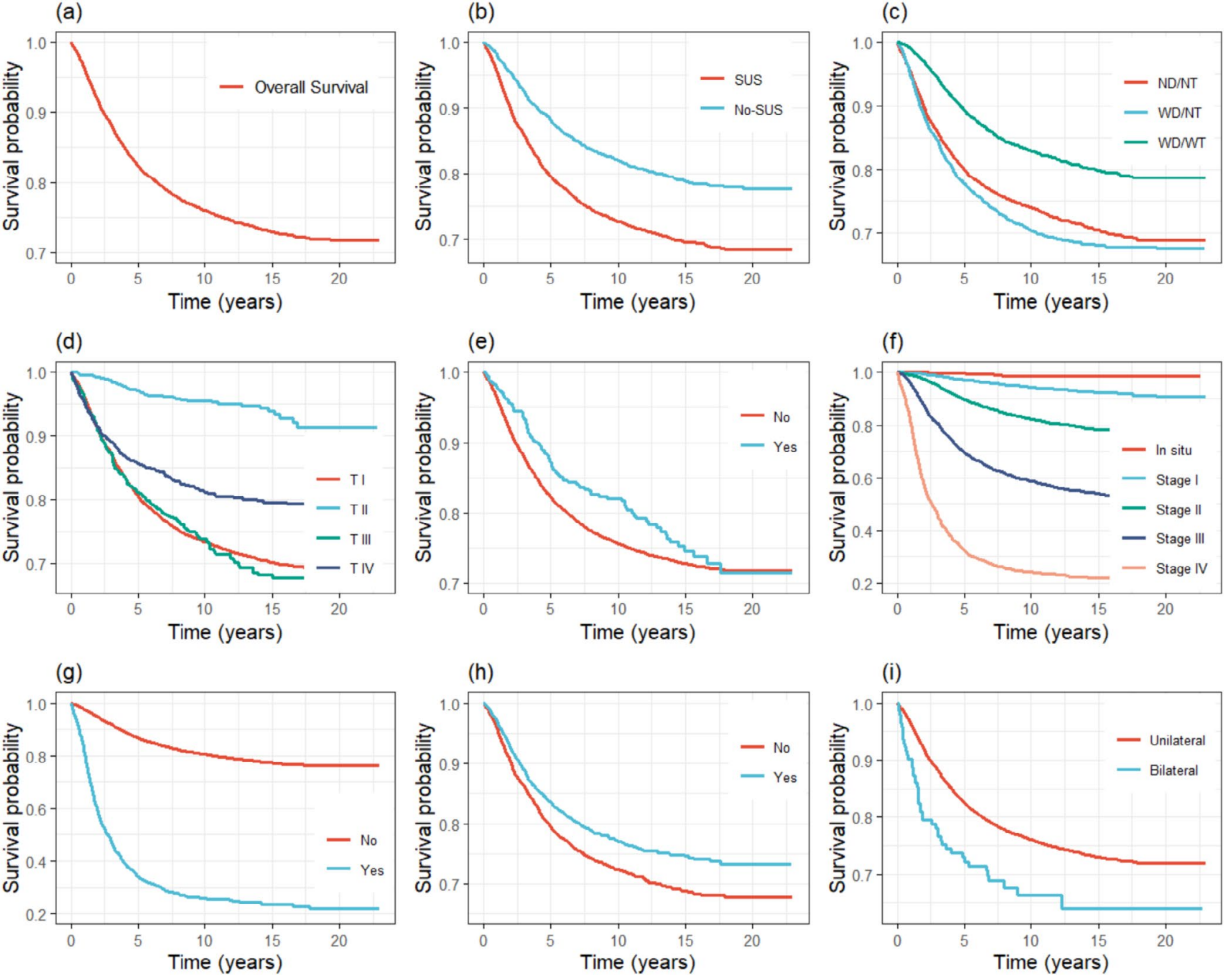
Kaplan–Meier specific survival curves for (a) all population, (b) forwarding source, (c) previous diagnosis and treatment (ND/NT: no diagnosis/no treatment; WD/NT: with diagnosis/no treatment; WD/WT: with diagnosis/with treatment), (d) histological type of primary tumor (TI: invasive carcinoma of no special type; TII: ductal carcinoma in situ; TIII: invasive lobular carcinoma; TIV: others), (e) occurrence of more than 1 primary tumor (Yes: > 1 primary tumor; No: 1 primary tumor), (f) clinical tumor staging by group (TNM), (g) distant metastasis, (h) family history of cancer, (i) tumor laterality.

Figure 1 depicts the 5‐year overall survival curves estimated by the Kaplan–Meier method for breast cancer‐specific mortality, for the entire sample and stratifications. Figure 1a depicts the overall survival of the sample at 82.4% (95% CI: 0.81–0.83).

**FIGURE 2:**
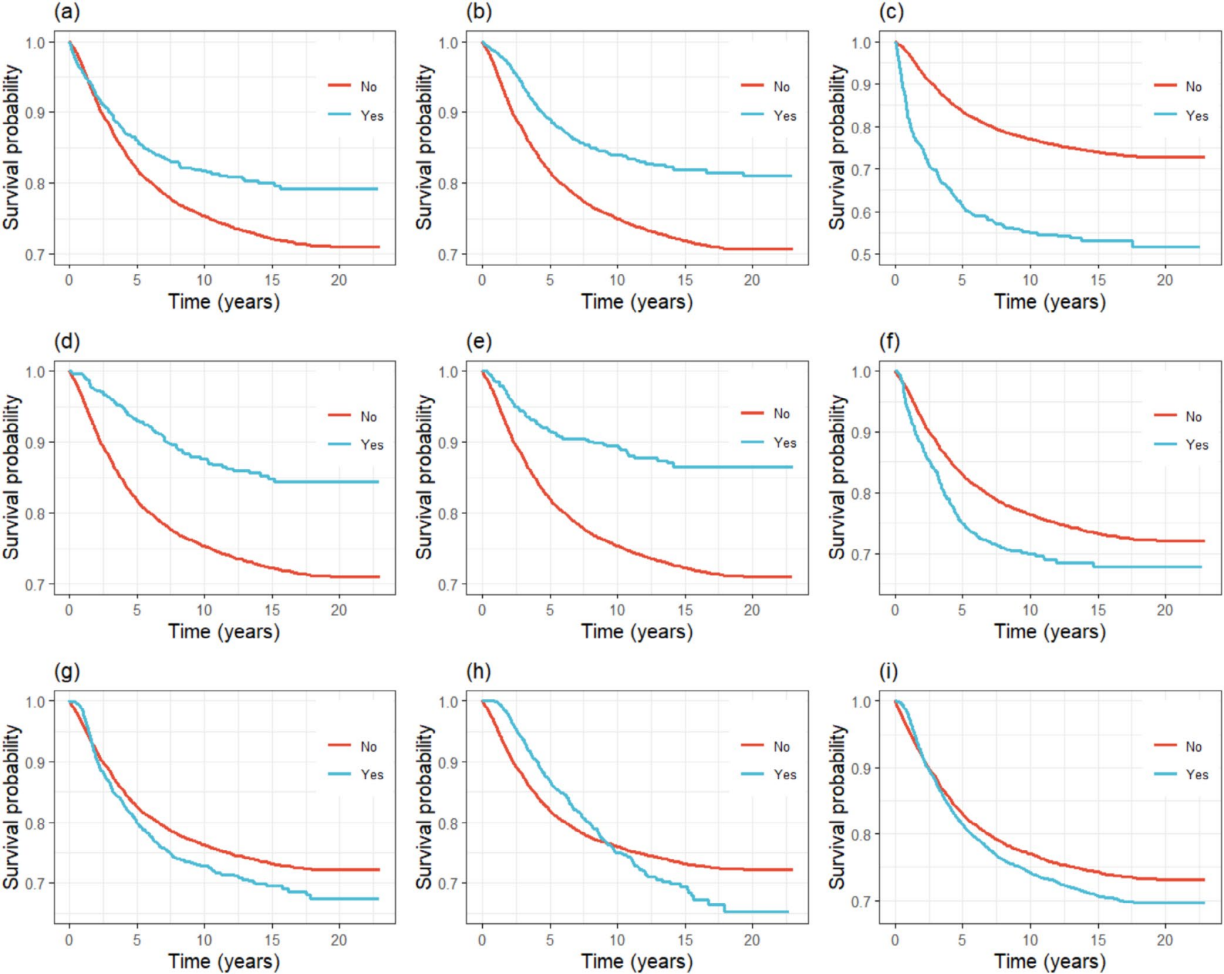
Kaplan–Meier specific survival curves for first treatment received at the hospital: (a) surgery (SX), (b) radiotherapy (RT), (c) chemotherapy (CT), (d) hormone therapy (HT), (e) SX + RT, (f) CT + SX, (g) SX + CT + RT, (h) SX + CT + RT + HT, and (i) other combinations.

Figure 2 demonstrates the 5‐year global survival curves estimated by the Kaplan–Meier method for breast cancer‐specific mortality, for the entire sample, and for all types of treatments.

The variables histologic type of the primary tumor, presence of more than one primary tumor, and first treatment received in the hospital (categories SX + CT + RT, SX + CT + RT + HT, and other combinations) did not meet the proportional hazards assumption over time. This violation was visually evident from the crossing of survival curves within the corresponding subgroups, as illustrated in Figures 1d,e, 2g–i. In addition, the variable family history of cancer exhibited a high number of missing observations, whereas tumor laterality showed marked imbalance between categories. Given these issues—both the violation of proportionality and the limitations related to data completeness or category distribution—these variables were not included in the multivariable Cox regression analysis.

Table 3 presents the results of the hazard ratio analyses based on the Cox multivariate regression model, which were conducted to evaluate the effects of multiple covariates on breast cancer survival (*n* = 7191). Women with breast cancer referred from the Private healthcare system had a significantly lower mortality risk than those referred from the Public healthcare system SUS (HR = 0.83; 95% CI: 0.75–0.93; *p* = 0.001). Moreover, women who had a previous diagnosis and treatment exhibited a 44% reduction in the risk of mortality compared to the group of women without a diagnosis and without treatment (HR = 0.66; 95% CI: 0.57–0.76; *p* < 0.001).

**TABLE 3:** Cox regression multivariate analysis of the risk factors of the specific mortality in patients with breast cancer (*n* = 7191).

Variables	HR	95% CI	*p* value
Forwarding source			
Public healthcare system (SUS)	Ref	—	—
Private healthcare system	0.83	0.75–0.93	0.001
Previous diagnosis and treatment			
No diagnosis/no treatment	Ref	—	—
With diagnosis/no treatment	0.95	0.85–1.06	0.373
With diagnosis/with treatment	0.66	0.57–0.76	< 0.001
Clinical tumor staging by group (TNM)			
Stage 0 (in situ)	0.15	0.05–0.42	< 0.001
Stage I	Ref	—	—
Stage II	2.78	2.25–3.43	< 0.001
Stage III	7.28	5.92–8.94	< 0.001
Stage IV	17.22	12.48–23.77	< 0.001
Distant metastasis			
No	Ref	—	—
Yes	1.49	1.14–1.94	0.003
Surgery (SX)			
No	Ref	—	—
Yes	0.83	0.68–1.01	0.072
Radiotherapy (RT)			
No	Ref	—	—
Yes	0.84	0.69–1.02	0.088
Chemotherapy (CT)			
No	Ref	—	—
Yes	1.24	1.05–1.474	0.010
Hormone therapy (HT)			
No	Ref	—	—
Yes	0.87	0.64–1.17	0.368
SX + RT			
No	Ref	—	—
Yes	0.89	0.62–1.28	0.547
CT + SX			
No	Ref	—	—
Yes	0.95	0.79–1.15	0.658

Tumor stage III (HR = 7.28; 95% CI: 5.92–8.94; *p* < 0.001) and IV (HR = 17.22; 95% CI: 12.48–23.77; *p* < 0.001) were associated with a 7.28‐fold and 17.22‐fold increased risk of specific mortality due to breast cancer, respectively, compared to stage I. Women with distant metastasis exhibited a 49% higher risk of mortality (HR = 1.49; 95% CI: 1.14–1.94; *p* = 0.003), compared to their peers. The results indicated that women who underwent chemotherapy alone exhibited a 24% increased risk of mortality (HR = 1.24; 95% CI: 1.05–1.47; *p* = 0.01), with this difference being statistically significant.

## 5 Discussion

This statewide analysis provides updated evidence on breast cancer–specific survival within the Oncology Care Network of Espírito Santo, leveraging a large real‐world dataset derived from linkage between hospital cancer registries and mortality records. The findings confirm substantial survival inequalities driven primarily by stage at diagnosis and by structural differences in referral pathways across the public and private healthcare systems, consistent with patterns described in Brazil and other middle income settings [8, 9, 23].

As in previous studies, advanced stage at diagnosis remained the strongest predictor of mortality [23, 24]. Women diagnosed with stage III–IV disease experienced substantially worse survival, reflecting persistent challenges in achieving timely diagnostic evaluation. Although screening history was unavailable, the stage distribution observed parallels reports documenting heterogeneous access to mammography, imaging, and diagnostic work‐up across Brazilian regions [25, 26].

A central contribution of this study is the evaluation of referral source—public (SUS) vs. private care—as a proxy of access pathways. Women referred from the private healthcare system exhibited lower mortality risk than those entering through SUS, even after adjusting for stage, metastatic status, age, education, and year of diagnosis. Similar public–private disparities have been reported in other Brazilian cohorts, where privately insured women experience shorter diagnostic intervals and improved survival [8, 27, 28, 29]. For instance, a referral‐center cohort in southeast Brazil found 5‐year survival of 80.6% in private care versus 68.5% in public care [8]. Moreover, in a large middle‐income country sample, treatment in private facilities was associated with better outcomes, likely reflecting more timely diagnosis and access to comprehensive care [30]. Ecological studies have also shown that lower access to cancer treatment services correlates with higher breast‐cancer mortality at the population level [31]. Finally, recent surveys among oncologists reveal systematic barriers in public settings—including delays in diagnostics, limited availability of therapies, and restricted access to specialized care—which may contribute to worse prognosis among public‐sector patients [32].

These patterns likely reflect broader socioeconomic gradients: individuals accessing private care typically have higher income, greater educational attainment, and fewer structural barriers to treatment initiation, factors that may introduce unmeasured confounding [33]. Although our analyses controlled for known prognostic variables, the possibility of residual confounding remains and is consistent with findings from other observational studies in Brazil [27, 34, 35]. Taken together, these findings support that structural inequities in access to diagnostics, treatment initiation, and specialized oncology services likely underlie the observed survival advantage for patients treated in the private health system.

Treatment variables also demonstrated significant associations with survival; however, their interpretation requires caution. In registry‐based studies, treatment receipt reflects availability and access rather than causal treatment effectiveness. Because treatment indications differ by stage, we adjusted all models for stage to minimize confounding by clinical eligibility. Nonetheless, immortal time bias may still occur when treatment timing is not incorporated—a limitation widely recognized in survival analyses using administrative data [36]. Accordingly, we interpret treatment variables as indicators of service access rather than measures of therapeutic benefit.

Another important finding concerns the large differences in early diagnosis and treatment initiation across municipalities and health service types. Such disparities reflect known structural challenges in Brazil's oncology network, including regional concentration of specialized services and fragmented referral pathways [11, 12, 13]. The statewide scope of this study made possible through HCR–SIM linkage highlights the need to strengthen early detection strategies and improve coordination between primary care, diagnostic services, and oncology units.

### 5.1 Strengths and Limitations

Strengths include the statewide cohort encompassing all treatment centers, deterministic linkage ensuring more accurate identification of breast cancer deaths, and the use of cause‐specific Cox modelling to address competing risks. This approach improves upon earlier studies in Espírito Santo, which were limited by incomplete mortality ascertainment.

However, several limitations should be acknowledged. First, the lack of data on comorbidities, income, and insurance status prevents adjustment for important confounders. While educational level and age were included, residual confounding may partially explain the survival differences between public and private pathways, as observed in previous Brazilian and international studies [33, 35]. Second, treatment timing was not analyzed, precluding correction for immortal time bias [36]. Third, staging information was missing for a subset of cases; because missingness was not at random, creating an “unknown stage” category would introduce misclassification bias. A complete‐case approach was therefore used in accordance with STROBE recommendations. Finally, screening and diagnostic intervals were unavailable, preventing examination of how early detection practices influence stage distribution.

Finally, treatment variables in the HCR were recorded only as categorical indicators of whether a modality was performed, without consistent or complete information on treatment initiation dates. Consequently, we were unable to model treatment as a time‐dependent exposure, and some degree of immortal time bias cannot be excluded. Because our primary objectives were not to evaluate treatment effectiveness, but rather to characterize prognostic factors such as referral source, stage, and metastatic status, the potential impact of this bias on the main findings is likely limited. Future studies should incorporate time‐dependent or landmark analytic approaches as more granular treatment‐timing data become available.

## 6 Conclusion

In this statewide cohort, breast cancer survival in Espírito Santo was strongly influenced by stage at diagnosis and access pathways. Women referred through the private healthcare system experienced significantly lower mortality than those referred from the public sector, reflecting system‐level inequalities in diagnostic timeliness and treatment opportunity. These findings underscore the need to strengthen early detection, improve referral coordination, and expand equitable access to high‐quality oncology care. Linkage between HCR and SIM is essential for ongoing survival surveillance and for guiding evidence‐based cancer control strategies in Brazil.

## Author Contributions

**Luís Carlos Lopes‐Júnior:** conceptualization, investigation, funding acquisition, writing – original draft, writing – review and editing, visualization, validation, methodology, software, formal analysis, project administration, resources, data curation, supervision.

## Funding

This study was financially supported by the Fundação de Amparo à Pesquisa e Inovação do Espírito Santo (FAPES), Call FAPES/CNPq/Decit‐SCTIE‐MS/SESA No. 09/2020‐PPSUS (Grant Agreement: 155/2021, Process Number: 2021‐F0436).

## Conflicts of Interest

The authors declare no conflicts of interest.

## Data Availability

The data that support the findings of this study are available on request from the corresponding author. The data are not publicly available due to privacy or ethical restrictions.
